# Bone mineral accrual and gain in skeletal width in pre-pubertal school children is independent of the mode of school transportation – one-year data from the prospective observational pediatric osteoporosis prevention (POP) study

**DOI:** 10.1186/1471-2474-8-66

**Published:** 2007-07-11

**Authors:** Gayani Alwis, Christian Linden, Magnus Dencker, Susanna Stenevi-Lundgren, Per Gardsell, Magnus K Karlsson

**Affiliations:** 1 Clinical and Molecular Osteoporosis Research Unit, Department of Clinical Sciences, Lund University, Sweden; 2 Department of Orthopaedics, Malmö University Hospital, SE-205 02 Malmö, Sweden; 3 Department of Clinical Physiology, Malmö University Hospital, SE-205 02 Malmö, Sweden

## Abstract

**Background:**

Walking and cycling to school could be an important regular source of physical activity in growing children. The aim of this 12 months prospective observational study was thus to evaluate the effect of self-transportation to school on bone mineral accrual and gain in bone width in pre-pubertal children, both traits independently contributing to bone strength.

**Methods:**

Ninety-seven girls and 133 boys aged 7–9 years were recruited as a part of the Malmö Pediatric Osteoporosis Prevention (POP) Study in order to evaluate the influence of self-selected school transportation for the accrual of bone mineral and bone width. Children who walked or cycled to school were compared with children who went by bus or car. Bone mineral content (BMC) was measured by dual energy X-ray absorptiometry (DXA) in the lumbar spine (L2–L4), third lumbar vertebra (L3) and hip, and bone width was calculated at L3 and femoral neck (FN). Changes during the first 12 months were compared between the groups. Subjective duration of physical activity was estimated by a questionnaire and objective level of everyday physical activity at follow-up by accelerometers worn for four consecutive days. All children remained in Tanner stage 1 throughout the study. Comparisons were made by independent student's *t*-tests between means, ANCOVA and Fisher's exact tests.

**Results:**

There were no differences in baseline or annual changes in BMC or bone width when the transportation groups were compared. No differences were detected in objectively measured daily level of physical activity by accelerometer. All children reached above 60 minutes of moderate to intense daily physical activity per day, the international recommended level of daily physical activity according to the United Kingdom Expert Consensus Group.

**Conclusion:**

The everyday physical activity in these pre-pubertal children seems to be so high that the school transportation contributes little to their total level of physical activity. As a result, the choice of school transportation seems not to influence the accrual of bone mineral or gain in bone size during a 12-month follow-up period.

## Background

Osteoporosis is a risk factor for fractures, a significant cause of morbidity and mortality in the elderly [1]. At present work is devoted to preventing low areal bone mineral density (aBMD). Over the past two decades, research has focused on whether modifiable lifestyle factors can optimize peak bone mass (PBM) as a 10% increase in PBM could delay osteoporosis by 13 years [2,3]. Among the key factors, physical activity has been described as a strategy to optimize skeletal development, as reports have inferred both highly intense [3,4] and moderately intense training [5-7] to increase the accrual of bone mineral. Trials also suggest that the skeletal benefits of exercise can be attained at a population level [8,9]. The school has then been regarded as one arena to launch such programs, as it is one of the few places where all children can be targeted [8,9]. Transportation mode to school has been described as another such possibility. Cross-sectional studies support this when reporting that walking and cycling to school are associated with a higher level of physical activity compared to traveling by vehicle [10,11]. However, to our knowledge no prospective studies have specifically evaluated the hypothesis.

The aim of this population-based cohort study in pre-pubertal Swedish children was to evaluate whether walking and cycling to school were associated with a higher level of physical activity and enhanced skeletal development during one year compared to traveling by car or bus.

## Methods

The Pediatric Osteoporosis Prevention (POP) Study is a prospective exercise intervention study launched in Malmö, Sweden, in 1999. As described [8,9], this trial was designed to annually assess musculo-skeletal development in children when they commence school. In the one-year report evaluating the skeletal effects of the exercise intervention program, 53 girls and 81 boys aged 7–9 years were assigned to a school-curriculum-based general physical activity program for 40 min/day during school (200 min/week). Fifty girls and 57 boys in the same ages assigned to the general school curriculum of physical activity (60 min/week) served as controls. Thirty-six of the boys and 21 of the girls spent more than two hours/week in organized spare-time sports activities. Since the current study aimed to evaluate the effects of school transportation mode, all children were pooled and divided into two groups: (1) those who walked or cycled to school and (2) those who traveled by car or bus. All, except one boy adopted from Colombia, were Caucasians without any disease or medication known to influence bone metabolism. As described in previous publications, no differences were found between the study participants and non-participants regarding height, weight or body mass index (BMI) [8,9].

A questionnaire evaluated lifestyle factors such as school transportation, diseases, medications and subjective estimated duration of physical activity as hours per week spent in organized physical activity. Six girls and 5 boys did not answer the question about mode of school transportation. Thus, this study includes data on 97 girls and 133 boys. Sixty girls, of whom 30 were registered in the intervention group and 30 in the control group, and 75 boys, of whom 34 were registered in the intervention group and 41 in the control group, walked or cycled to school. Thirty-seven girls, of whom 18 were registered in the intervention group and 19 in the control group, and 58 boys, of whom 43 were registered in the intervention group and 15 in the control group, traveled by car or bus. The children who walked and cycled did so with their own choice of intensity.

Bone mineral content (BMC, g) at the lumbar spine (L2–L4 vertebrae), third lumbar vertebra (L3) and the hip (femoral neck [FN], Ward's region and the trochanter) and total body lean mass and fat mass were measured by dual-energy X-ray absorptiometry (DXA, DPX-L version 1.3z, Lunar^®^, Madison, WI). The width of the L3 vertebra and the FN were estimated from the spine and the hip scans. The coefficient of variations (CV), which was evaluated by duplicate measurements in 13 healthy children aged 7–15 years, was found to be 1.4–3.7% for BMC, 2.2% for L3 width, 3.7% for total body fat mass and 1.5% for total body lean mass. Calibration of the machine was done every day with the Lunar^®^phantom. The research technicians performed all the measurements and analyzed all the scans. Body weight was measured to the nearest 0.1 kg with an electric scale and body height to the nearest 0.5 cm by a wall-tapered height meter. All children remained in Tanner stage 1 throughout the study as assessed by the research nurse [12].

The methodology of physical activity measurement has previously been presented in detail [13-15]. Physical activity was assessed using the MTI (Manufacturing Technology Incorporated, Fort Walton Beach, FL, USA) accelerometer, model 7164 for four consecutive days. Accelerometer data are averaged over a period called an epoch. A recording epoch of ten seconds was selected for this study. SAS-based software (SAS Institute Inc, Cary, NC, USA) was used to analyze all accelerometer data. This software automatically deletes missing data, defined as continuous sequences of 60 consecutive epochs (i.e. 10 minutes) or more with zero counts. This was done based on the assumption that all such sequences of zeros lasting longer than ten minutes were caused by the accelerometer not being worn. In order to minimize inter-instrumental variation, all accelerometers were calibrated against a standardized vertical movement. Mean activity was considered to be the total accelerometer counts per valid minute of monitoring (mean counts/min). Time spent performing above 3 METs was considered to reflect moderate-to-vigorous activity (MVPA), and time spent above 6 METs was considered to reflect vigorous activity (VPA). Cut-off points used for all children were >167 counts/epoch for MVPA and >583 counts/epoch for VPA [16,17]. The proportion of children that reached current health-related physical activity recommendations was also calculated. We used the United Kingdom Expert Consensus Group recommendation that children accumulate at least 60 minutes of moderate to intense activity per day [18].

Baseline measurements were performed at the commencement of school, with follow-up evaluations during the same months one year later in the group with extra training classes and two years later in the control group. We accepted this difference as data in the literature have reported that growth occurs in a linear fashion during the pre-pubertal period [19-28]. For all data, we calculated and compared the changes per 365 days.

Informed written consent was obtained from parents or guardians of participants before the study start. The study was approved by the Ethics Committee of Lund University (LU 453-98; 1998-09-15), Sweden, and conducted according to the Helsinki Declaration of 2000.

Statistical calculations were performed with Statistica^®^, version 6.1 (StatWin^®^). Data are presented as means (SD). Absolute annual changes were calculated as changes per 365 days. The girls and boys who walked or cycled to school were compared with those who traveled by bus or car with two-tailed tests; independent student's *t*-tests between means and Fisher's exact test. Analysis of covariance (ANCOVA) was also used to adjust for duration of organized physical activity if the children were in the intervention or control group. A *p*-value of < 0.05 was considered as a statistically significant difference.

## Results

No significant differences were found at baseline for any of the lifestyle factors, physical activity, age, height, weight, body composition, BMC or bone width when girls and boys who walked or cycled to school were compared with those who traveled by car or bus (Table 1 and 2). These results remained after adjustment for duration of organized physical activity.

**Table 1:** Lifestyle factors in children who walked and cycled or traveled by car or bus to school. Data are presented as numbers of children with the proportion within each group (in brackets) expressed as % or as mean (SD).

	**Girls (n = 97)**			**Boys (n = 133)**		
	
	**Walking or cycling**	**Car or bus**	***p*-value**	**Walking or cycling**	**Car or bus**	***p*-value**
**At baseline**						
Numbers	n = 60	n = 37		n = 75	n = 58	
Distance to school (km)	0.5 (0.5)	1.7 (1.9)	*p *< 0.001	0.7 (0.64)	1.6 (1.2)	*p *< 0.001
Excluding dairy products	0 (0%)	0 (0%)	1.0	0 (0%)	0 (0%)	1.0
Drinking coffee	1 (2%)	0 (0%)	1.0	1 (1%)	1 (2%)	1.0
Tried to lose weight	1 (2%)	0 (0%)	1.0	0 (0%)	0 (0%)	1.0
Chronic disease	2 (3%)	1 (3%)	1.0	6 (8%)	3 (5%)	0.73
Medication	2 (3%)	5 (14%)	0.10	8 (11%)	5 (9%)	0.78
Fractures	11 (18%)	2 (5%)	0.12	9 (12%)	3 (5%)	0.23
Total physical activity (hour per week)	0.8 (1.0)	1.2 (1.8)	0.17	1.3 (1.1)	1.7 (1.4)	0.10
						
**During study period**						
Total physical activity (hour per week)	3.7 (1.7)	3.5 (2.1)	0.53	4.0 (2.1)	5.0 (2.0)	*p *< 0.01
						
**At follow-up – Accelerometer data**						
Numbers	n = 53	n = 34		n = 68	n = 54	
Recording time per day (hours/day)	11.9 (1.3)	11.9 (1.3)	0.97	12.0 (1.4)	11.9 (1.4)	0.83
Mean activity (mean counts/min)	614 (148)	629 (164)	0.66	728 (234)	758(257)	0.51
Moderate to vigorous activity (min/day	188 (35)	195 (42)	0.39	207 (52)	211 (47)	0.68
Vigorous activity (min/day)	34 (13)	36 (14)	0.55	45 (20)	46 (20)	0.84

**Table 2:** Baseline data and twelve-month changes, evaluating the effects of school transportation mode. Anthropometrics, bone mineral content (BMC) and bone width are included. Data are presented as means (SD).

	Girls (N = 97)						Boys (N= 133)					
	
	Baseline			Annual changes			Baseline			Annual changes		
	Walking or cycling	Car or bus	*p*-value	Walking or cycling	Car or bus	*p*-value	Walking or cycling	Car or bus	*p*-value	Walking or cycling	Car or bus	*p- *value
Numbers	N = 60	N = 37		N = 60	N = 37		N = 75	N = 58		N = 75	N = 58	

Age (yrs)	7.8 (0.6)	7.7 (0.6)	0.42	----	----	----	7.9 (0.6)	7.9 (0.6)	0.83	-----	----	----
Weight (kg)	27.5 (5.4)	27.3 (5.3)	0.90	3.3 (1.7)	3.5 (2.1)	0.60	28.0 (5.4)	28.2 (6.1)	0.89	3.3 (1.2)	3.1 (1.9)	0.43
Height (cm)	128.6 (6.9)	128.8 (6.3)	0.87	5.8 (1.0)	5.9 (1.2)	0.50	130.0 (6.9)	129.2 (6.3)	0.52	5.7 (0.8)	5.5 (1.0)	0.20
Lean mass (kg)	20.1 (2.6)	20.0 (2.4)	0.84	2.1 (0.7)	2.0 (0.7)	0.48	22.0 (3.1)	21.5 (3.0)	0.32	2.3 (0.5)	2.1 (0.6)	0.06
Fat mass (kg)	5.1 (3.6)	5.2 (3.1)	0.98	1.4 (1.2)	1.6 (1.6)	0.54	3.9 (3.0)	4.3 (3.7)	0.50	1.1 (1.0)	1.3 (1.6)	0.50
												
**BMC (g)**												
L2–L4	15.4 (3.0)	15.3 (3.5)	0.89	2.0 (0.9)	2.2 (1.2)	0.36	16.1 (2.9)	15.9 (3.7)	0.73	2.2 (1.1)	2.4 (1.2)	0.28
Third lumbar vertebra	5.1 (1.0)	5.2 (1.3)	0.87	0.69 (0.4)	0.78 (0.7)	0.41	5.5 (1.0)	5.3 (1.3)	0.36	0.70 (0.6)	0.88 (0.7)	0.12
Femoral neck	2.6 (0.7)	2.6 (0.5)	0.68	0.29 (0.7)	0.37 (0.5)	0.57	2.9 (0.7)	2.9 (0.7)	0.53	0.32 (0.4)	0.26 (0.4)	0.42
Ward	1.2 (0.5)	1.1 (0.5)	0.65	0.17 (0.6)	0.19 (0.5)	0.85	1.3 (0.5)	1.3 (0.4)	0.47	0.17 (0.3)	0.15 (0.3)	0.68
Trochanter	2.7 (1.3)	2.6 (0.9)	0.83	0.66 (0.9)	0.66 (0.9)	0.97	2.8 (1.5)	2.7 (1.2)	0.63	0.55 (0.7)	0.48 (0.7)	0.56
												
**Width (cm)**												
Third lumbar vertebra	2.89 (0.27)	2.88 (0.23)	0.88	0.12 (0.10)	0.13 (0.11)	0.70	3.11 (0.23)	3.03 (0.30)	0.13	0.10 (0.11)	0.13 (0.16)	0.16
Femoral neck	2.47 (0.29)	2.43 (0.26)	0.42	0.13 (0.28)	0.13 (0.25)	0.95	2.50 (0.25)	2.45 (0.21)	0.23	0.14 (0.15)	0.11 (0.18)	0.38

During the follow-up period, the gain in height, weight, body composition, BMC or bone width did not differ between the two groups. These results remained after adjustment for subjective estimated duration of organized physical activity during the study period and whether the children had extra physical education classes or not. Furthermore, there were no gender differences in the annual skeletal changes (p = 0.07–1.00), data not shown.

The objective measured level of physical activity (accelerometer) at follow-up was no different for children who walked or cycled to school compared with those who traveled by car or bus (Table 1). Furthermore, as published [13], the objective registration revealed that all children fulfilled the international recommended level of 60 minutes of moderate to intense physical activity per day [18].

## Discussion

In this cohort, there were no additional benefits in skeletal development in children who walked or cycled to school compared to children who traveled by car or bus. It must be emphasized that this report only evaluates the skeleton and not other health-related effects induced by physically active school transportation. Furthermore, our finding that a physically active mode of transport to school was not associated with higher overall levels of physical activity contrasts with the results from previous studies [10,11]. In fact, in our study the boys who traveled by car or bus had a higher level of self-reported physical activity throughout the study than the boys who walked or cycled (Table 1). While the reason(s) for this finding are not clear, when we analyzed the accelerometer data at follow-up we found that there were no differences in activity levels between the two groups of boys.

The finding that the mode of school transportation did not confer any additional benefits to the accrual of bone mineral and gain in bone width could be explained by a number of factors. First, the children in this study had relatively high levels of physical activity (Table 1). This is supported by the accelerometer data, which revealed that all children reached the international level of 60 minutes per day of physical activity [13], set by the United Kingdom Expert Consensus Group [18], independent of school transportation mode. Second, the distance from home to school was only 0.5 to 1.7 km, and thus the overall contribution of walking or cycling to school to each child's overall level of physical activity was likely to be relatively insignificant. In support of these findings, Metcalf et al reported that walking to school was not associated with overall levels of physical activity in children aged 5 [29]. In view of this, it must also be emphasized that cycling or walking to school provides only a short duration of low-intense activity per day [30,31] and that this low-impact stimulus with a relatively short duration could perhaps not be expected to lead to skeletal effects of biological significance. Finally, it is likely that walking and cycling to school did not provide a sufficient stimulus to overload bones and thereby cause an osteogenic response. Most previous studies have shown that weight-bearing exercise which is relatively high in magnitude, applied at a high rate and/or unusual or diverse in nature (e.g. hopping, skipping, jumping), is necessary to enhance bone mineral accrual [6]. However, even osteogenic training provides small skeletal benefits compared to growth in pre-pubertal boys [5,32]. This view is further supported in a prospective 3-year report, inferring that boys on soccer exercise achieved only minor skeletal benefits in femoral BMC and BMD [33], even if a rather osteogenic sport was advocated [34]. However, as the general level of physical activity in older children is sometimes reported to be lower than in younger children [35,36], transportation mode could be important in these ages as well as in geographic areas with a long distance from home to school.

### Study strength

One strength of this prospective population-based design, increasing our ability to draw generalized inferences, and despite not being a randomized controlled trial, is that the similarity between the groups at baseline provides a higher level of evidence as regards the effect of school transportation than previously published cross-sectional studies. The similarity in the anthropometric characteristics between individuals who did or did not participate in this study provides further support that our inferences could be generalized. The use of accelerometers, as an objective estimate of daily physical activity, is also positive compared to subjective estimates of physical activity [37,38]. Finally, this study included a relatively large sample of children who either walked/cycled or traveled to school by bus/car when compared to most previous reports [39-41].

### Study limitations

This study evaluates children aged 7–9 years. This could lead to problems, as there are reports suggesting that the skeleton is not responsive to physical load until Tanner stages 2 and 3 are reached [42,43]. Yet this view is opposed by prospective reports, inferring that also children in Tanner stage 1 may attain benefits with mechanical load [5,32], actually reported in the cohort evaluated in this trial [9,44]. Furthermore, when the children in this cohort reach a higher age they increase their duration of spare-time physical activity [44,45]. That is, physically active school transportation will contribute a larger proportion of the total weekly amount of physical activity in young children. And beneficial effects achieved by physically active school transportation would then possibly be easier to capture in young children, due to the dose-response effect between exercise and bone mass. This is the reason why we focused on 7–9-year-old children.

The transportation classification was also based on the questionnaire, that is, no objective classification was made. The transportation mode was self-selected, that is, no randomization was done, which introduces a risk of selection bias. However, this must be regarded as a minor risk since there were no registered differences between the groups at baseline. Furthermore, the finding that the choice of transportation to school was more dependent on the distance to school than on the phenotype of the child, and that the drop-out analyses revealed no differences between participants and non-participants, strengthens the view that the inferences could be generalized. It would also have been an advantage if all four transportation modes had been comparable. However, due to the high risk of arriving at a type II error, this was not done. The study design, with half of the children having 40 min/day of extra physical education, could have also influenced our findings. But, as there were children with different transportation modes among the children with extra training classes and children without, and as the results remained after adjustment for being in extra school training or not, this risk is regarded as minimal. Finally, the one-year follow-up in the children with extra physical classes and the two-year follow-up in the control group could represent a source of error. However, this seems improbable as all results were reported as annual changes, and all children remained in Tanner stage I, and previous research has shown that growth occurs in a linear fashion during these years [19-28]. Furthermore, as the results remained, when extra physical classes were introduced (one-year follow-up) or not (two-year follow-up) as a covariate, this indicates that the problem did not interfere with the data. The study design has also previously been accepted [8,9]. Finally, this study was limited to a 12-month follow-up and it is possible that a study with a longer duration could detect a beneficial effect of school transportation mode on bone health.

## Conclusion

The everyday physical activity in 7–9-year-old Swedish children seems to be so high that school transportation does not contribute significantly to their total level of physical activity. As a result, the mode of transport to school seems not to have a significant effect on the accrual of bone mineral or gain in bone width over 12 months.

## Competing interests

The author(s) declare that they have no competing interests.

## Authors' contributions

GA was involved in the statistical analysis, the interpretation of data and the writing of the manuscript, CL and SSL collected all data except the accelerometer data and were involved in the drafting of the manuscript, MD collected the accelerometer data and was involved in the drafting of the manuscript, PG designed the study and was involved in collecting the data and MK designed the study, worked with the analysis and the interpretation of data and was in charge of writing the manuscript. All authors contributed intellectually to the manuscript and all authors have read the manuscript and approved the submission.

## Pre-publication history

The pre-publication history for this paper can be accessed here:


